# The Student’s Guide to Diseases of the Eye

**Published:** 1887-12

**Authors:** 


THE STUDENT'S GUIDE TO DISEASES OF THE EYE. By Edward Nettleship, F. R. C. S., with a chapter on Examination for Color Perception, by William Thomson, M. D., Philadelphia. Lea Bros. & Co. 1887. Price $2.00.

That this is the fourth edition, since 1879, of this book speaks well for its popularity. It has been thoroughly revised to date. The chapter on examination for color perception, by Dr. Thomson, is of much importance. We commend the book to the practitioner as well as the student.

